# Molecular Aspects of Breast Cancer Metastasis to the Brain

**DOI:** 10.4061/2011/219189

**Published:** 2011-11-29

**Authors:** Jodi M. Saunus, Majid Momeny, Peter T. Simpson, Sunil R. Lakhani, Leonard Da Silva

**Affiliations:** ^1^UQ Centre for Clinical Research, The University of Queensland, Herston, QLD 4029, Australia; ^2^School of Medicine, The University of Queensland, Herston, QLD 4029, Australia; ^3^Pathology Queensland, The Royal Brisbane and Women's Hospital, Herston, QLD 4029, Australia

## Abstract

Our knowledge of the biology underlying the development of brain metastases (BM) from breast cancer has improved over the last decade due to large clinical epidemiological studies, animal models of metastasis, and the use of high-resolution gene expression profiling technologies. However, there are still major gaps in our understanding of the mechanisms utilized by breast cancer cells to colonize the brain microenvironment, thus our arsenal of therapies remains relatively nonspecific, and the prognosis for breast cancer patients with BM remains poor. Additional insights into these mechanisms are necessary to facilitate the development of new preventive and curative therapeutic regimens to block this fatal disease. This paper aims to provide a general overview for the readers of what has been achieved in this field of research and its translation into clinical practice to date and to highlight exciting new areas of research that promise to inform the development of new targeted therapies for BM.

## 1. An Overview of Metastasis

Metastasis, or metastatic disease, is the spread of cancer cells from one organ to a distant site via the blood or lymph. In the Nineteenth Century, Paget asked whether the distribution of metastases in different organs was simply a matter of chance. He studied autopsy records of women with breast cancer, revealing a nonrandom pattern of metastatic colonization. He proposed a hypothesis that tumour cells (the “seed”) could have specific affinity for the microenvironments of certain organs (the “soil”) [1]. This intriguing phenomenon is called organotropism, and Paget's hypothesis has now been repeatedly substantiated, with growing recognition of the importance of tumour cell interactions with the stromal microenvironment in supporting the establishment of metastases (see below). For instance, analysis of large autopsy series has showed that lung, breast, melanoma, renal, and colon cancers are the most common primary tumours to metastasize to the brain [2, 3]. There is a theory that the primary tumour could contribute to priming “premetastatic niches” prior to the establishment of micromestastases, thereby influencing organ tropism. Kaplan, Lyden, and colleagues' analysis of mouse models of lung metastasis implicated certain chemokines in this process, as well as mobilisation of haematopoietic precursor cells to pre-metastatic sites [4], however the mechanisms underlying creation of pre-metastatic niches are not fully understood [5].

The metastatic process is very inefficient. In order to accomplish distant metastasis, tumour cells must first detach and/or escape from the primary site, then survive as circulating tumour cells (CTCs) in the absence of the microenvironment cues with which they were conceived. Most CTCs are cleared from initial trapping sites within a few days. Those that survive, and succeed in extravasating, engraft at a distant site forming a micrometastasis, then may proliferate to form a clinically significant lesion after a fairly unpredictable period of latency (dormancy) spent meeting requirements for cell division in the new microenvironment [6, 7]. The poorly understood “dormancy” phenomenon poses a major challenge in metastasis management. Despite being attractive drug targets (at least conceptually), CTCs and micrometastases are undetectable with current hematologic and imaging technologies and are thought to be insensitive to chemotherapeutics that target rapidly dividing cells. 

It is now becoming clear that only a very small proportion of primary tumour cells are capable of forming clinically significant metastatic lesion [8]. Because these cells share critical features with normal stem cells (namely, multi-lineage potential in reseeding a secondary tumour and self-renewal), they have been called cancer stem cells (CSCs). It is hypothesized that a complex combination of tumour cell intrinsic and extrinsic processes eventually culminates in the activation of dormant CSC proliferation at distant sites [8]. Current evidence suggests that this includes continuing tumour cell genetic evolution, adaptation, and selection of those cells with signaling programs that best engage and exploit the new microenvironment [9, 10]. Observations on the temporal courses of metastasis, from different primary tumour types, provide some insight into the biology underlying these processes. For example, subsets of breast and lung carcinomas show similar overall organ tropism (brain, bone, lung, and liver), but strikingly different progression times, with distant relapse detected early in lung cancer (macrometastases established within months of diagnosis), and relatively late in breast cancer progression (after years to decades of remission). This suggests early acquisition of metastasis-enabling genetic alterations in lung cancer, in contrast to a longer latency period as CTCs and/or micrometastases in breast cancer. 

At least three categories of metastasis genes have been proposed to facilitate the multistep metastatic cascade (reviewed in [11]): (1) “initiation” genes that facilitate detachment (e.g.,* CDH2* (encodes N-Cadherin) and *TWIST)*, extracellular matrix degradation (e.g., *MMP*s) or angiogenesis (e.g., *VEGF*); (2) “progression” genes (e.g., *PTGS2 *(encodes COX-2) and *MMP-1) *that regulate extravasation of circulating tumour cells and are involved in metastatic colonisation; (3) “virulence” genes (e.g., *IL6* and *TNF*α**), which promote survival in circulation, and/or provide a proliferative advantage in the distant microenvironment. Apart from these metastasis-promoting genes, there is a well-distinguished class of metastasis “suppressor” genes that represses tumour cell dissemination without any effect on primary tumour growth, including *KAI-1*, *BRSM1,* and *NME1 *[11]. These findings are mainly derived from studies using animal models. Historically, it has been difficult to understand the genetic alterations underlying metastasis by direct analysis of human tissue. Transcriptomic profiling of matched pairs of primary tumours and their metastases has demonstrated a high degree of similarity with minor differential gene expression [12–14]. However, the more recent application of next-generation whole genome sequencing (WGS) technologies is revealing subtle, but significant distinctions between metastases and their primary tumours of origin [15, 16] that were previously undetectable.

## 2. What Is the Impact of Brain Metastasis in the Natural History of Breast Cancer?

The prevalence of BM during the course of breast cancer disease has been reported to range from 10%–16%, reaching 30% when autopsy diagnoses of BM are included [17, 18]. Several factors have been reported to be associated with a higher risk of developing BM: patients less than fifty years old, four or more axillary lymph nodes involved with metastatic disease, basal phenotype, and high tumour grade [19–22]. Current therapeutic strategies for BM include whole brain radiation therapy (WBRT; the treatment mainstay since the 1950s), stereotactic radiosurgery, or surgery combined with radiotherapy. The median survival in untreated breast cancer patients with symptomatic BM is less than one month, 6–8 weeks in patients treated with steroids alone and 3–6 months when treated with WBRT [3, 23]. 

Patients with HER2-positive breast tumours are now also regarded to be at high risk for developing BM. Improved control of systemic disease with the anti-HER2 monoclonal antibody combined with poor BBB penetration have been regarded as culprits of this increased metastatic trend [24]. BM treatment strategies are currently informed by the histopathology of the primary tumour, and BM are rarely biopsied. Whilst many features are shared, it is becoming clear that metastases are distinct in their genetic landscape and expression of critical disease markers [12, 14–16]. Hence, future therapeutic development is likely to be based on features of the metastases themselves.

## 3. Does the Incidence of BM Correlate with Breast Cancer Molecular Subtypes?

**Figure 1:**
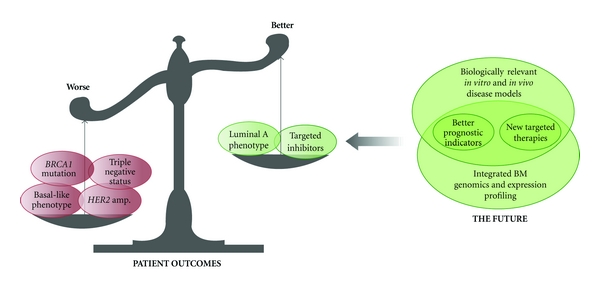
Summary of the major factors that increase or decrease risk of developing brain metastases in breast cancer and exciting new areas of research that promise to deliver the knowledge required for new targeted therapies and better prognostication.

Breast cancer is a heterogeneous disease with respect to molecular features perhaps best exemplified by the molecular subgroups identified by gene expression profiling including basal-like, luminal A (hormone receptor positive), luminal B, and HER2 amplified/over-expressed (HER2+) subtypes. These molecular subtypes are associated with different outcomes. It is generally accepted that the basal-like, luminal B and HER2+ subtypes are associated with particularly poor prognosis compared with the hormone receptor-positive, luminal A subtype [25, 26] (Figure 1). Basal-like tumours are generally high grade, have central areas of necrosis, invasive pushing borders and are characterized by the expression of markers including high-molecular weight cytokeratins (e.g., CK14 and CK5/6), p53 and the myoepithelial markers Smooth Muscle Actin (SMA) and p63 [27, 28]. 

We, and other groups, have reported a greater propensity of primary breast cancer with a basal-like immunophenotype to metastasize to the brain [20–22]. Moreover, patients with germline *BRCA1* mutations who develop breast cancer have a higher incidence of BM compared to germline *BRCA2* carriers and non-*BRCA1/2* patients [29]. These tumours have morphological similarities with those from the sporadic basal-like group [27, 28]. Patients with HER2+ tumours have also been shown to have an increased incidence of BM [30]. These metastatic patterns were further validated by a large study analysing, 3,726 cases, with a median follow-up of 14.8 years [31]. This work confirmed a higher rate of BM in HER2+-enriched and basal-like, compared to luminal A tumours. The cumulative incidence for BM in basal-like and HER2+ tumours were highest in the first five years after diagnosis, plateauing thereafter.

## 4. Current Understanding of BM from Breast Cancer Mechanisms

Gene expression profiling of breast tumours coupled with outcome data, functional analyses on cell lines, and *in vivo* animal models have shed light on our understanding of the colonization of the brain parenchyma by breast cancer cells [10, 11, 16, 42–44] (Figure 1).

Regarding the brain microenvironment, there are two main cell types in the neural tissue: neurons and glial cells, including microglia, astroglia, and oligodendroglia. There is a body of evidence suggesting that metastatic tumour cell interactions with the brain microenvironment facilitate the colonization process [43]. Interactions between breast cancer cells and pericytes and/or astrocytes might be responsible for alterations in the BBB and thus, development of BM from breast cancer. For example, Mendes et al. [45] reported that astrocyte-induced factors activated the ERK1/2 signalling pathway in rat mammary adenocarcinoma ENU1564 cells and thus enhanced the invasive features of these cells through increased expression of MMP-2. Furthermore, transfection of ENU1564 cells with TIMP-2, a natural inhibitor of MMP-2, reduced the *in vitro* invasive characteristics of these cells. BM was not observed in animals inoculated with ENU1564-TIMP-2, which implies a cardinal role for MMP-2 in BMBC [45]. In addition, reactive glia under coculture with breast cancer cell line MDA-MB-231 was shown to enhance growth of this same cell line [46]. Furthermore, the 435-Br1 cell line, which was derived from BM in a nude mouse, showed increased adhesion to astrocytes and enhanced growth *in vitro* in the presence of an astrocyte-conditioned media when compared to parental MDA-MB-435 breast cancer cells and the lung metastasis-derived variant 435-Lung2 [47].  Table 1 summarizes BM-associated genes identified in integrated studies that used combinations of *in vitro* cell culture-based functional assays, *in vivo* mouse xenograft models and the analysis of human clinical samples.

Palmieri et al. demonstrated that when human MDA-MB-231-BR cells, the brain metastatic derivative of the MDA-MB-231 cell line, were transfected with HER2, the HER2-overexpressing clones showed a threefold increase in the number of large BM compared with control MDA-MB-231-BR cells [39]. These findings are consistent with epidemiological studies showing increased incidence of BM in HER2+ breast cancer patients [30]. The same group showed *in vitro* that Lapatinib inhibited the phosphorylation of EGFR, HER2, and downstream signalling proteins leading to reduced proliferation and migration in 231-BR. This *in vitro* data was also replicated in a mouse model showing Lapatinib inhibited the growth of brain macrometastases seeded from EGFR-overexpressing 231-BR cells [48].

Another example of an integrated approach to understanding BM from breast cancer was published by Bos et al. In this study, cyclooxygenase, the epidermal growth factor receptor ligand HBEGF and *α*2,6-sialyltransferase acted as mediators of cancer cell passage through the BBB. Again, these findings were derived from *in vitro* functional analyses and animal models, with translation in clinical samples where meta-analysis of gene expression profiling of patients who had brain metastases and survival data were combined. The presence of this signature was related to shorter brain-metastasis free survival [34].

Several studies focusing on the functional significance of single genes have also contributed to our current understanding of BM development. In an effort to develop an experimental model of BM from breast cancer, Kim et al. used internal carotid artery injection of breast cancer cells into nude mice, which resulted in formation of different brain metastatic variant cell lines. These variants expressed higher levels of vascular endothelial growth factor (*VEGF*) and IL-8 than the nonbrain metastatic clones, suggesting a possible role for VEGF in BM from breast cancer [40]. Consistent with this, suppression of BM and induction of apoptosis were observed following treatment of the mice with the VEGF-receptor tyrosine kinase inhibitor, PTK787/Z 222584 [40]. A second study by Palmieri et al. analyzed gene expression profiles of laser-captured epithelial cells from BM of breast cancer patients compared with unmatched primary breast tumors. The results showed that hexokinase 2 (*HK2*), a critical enzyme in glucose metabolism, is increased in BM [49]. Increased expression of *HK2* was observed in the 231-BR brain metastastic breast cancer cell line [49]. A third example is the study by Chiu et al., which showed augmented expression of the activated form of signal transducer and activator of transcription 3 (*STAT3*) coupled with downregulation of Caveolin-1 (the structural component of caveolae involved in membrane trafficking and cell signaling) in BM compared with primary breast tumours. Furthermore, they showed that ectopic expression of Caveolin-1 or knockdown of *STAT3* reduces the invasive features of breast cancer cells *in vitro* and brain colonization *in vivo* [38].

Our group has combined gene expression array profiling, targeted somatic mutation analysis, and immunohistochemical profiling of BM from breast cancer and integrated the data to demonstrate activation of signaling pathways associated with the HER receptor family in BM compared to their matched primary breast tumours. Critically, the data showed an increase in *HER3* expression in breast cancer cells isolated from BM compared to matched primary tumours [12]. Neuregulin 1, the ligand for this receptor, is abundantly expressed in the brain [50] and is activated by a variety of stimuli, including hypoxia [51]. Consistent with this, we observed increased expression of hypoxia-inducible Factor 1*α* (*HIF-1*α*)* in the BM, likely reflecting the local hypoxic environment. Increased *HER3* expression has also been reported in BM from lung cancer [52]. These two clinical snapshots could reflect environmental selection and therefore adaptation of metastatic cells to the brain microenvironment, exploiting the Neuregulin-HER3 axis in order to succeed.

While there is ample evidence that miRNAs have determinant roles in tumour cell dissemination, the possible roles of these “micromanagers of metastasis” in BM from breast cancer are poorly understood. Using miRanda for target prediction, Zhang et al. showed that miRNA-1258 targets heparanase (HPSE) which is the dominant endoglycosidase in mammals. HPSE favors tumor cell spread through dissolution of the extracellular matrix. In addition, this study introduced an inverse association between the miR-1258 levels and heparanase expression and enzymatic activity [53]. These findings were confirmed when the authors compared miRNA-1258 levels in clinical samples of invasive ductal carcinoma and BMBC compared with the corresponding normal or primary tissues. Moreover, the expression of miR-1258 in BM from breast cancer cells has been shown to suppress heparanase *in vitro* cell invasion and experimental BM.

## 5. The Blood-Brain-Barrier Is an Additional Challenge for BM Therapeutic Development

The BBB comprises a specialized endothelium surrounded by a thick basement membrane and astrocytic endfeet [48]. Compared with endothelial cells from other vascular beds, brain microvascular endothelial cells characteristically have very low permeability to solutes and hydrophilic molecules, high electrical resistance, complex tight junctions, and an array of metabolic and transport systems that supply the brain with nutrients and eliminate brain metabolic by-products. The low permeability is important in protecting the brain from circulating toxins and restricting the migration of leukocytes and monocytes, consequently it is also hindrance to drug access. The BBB endothelia specifically express efflux transporters that further diminish the brain availability of certain chemotherapeutic agents, including p-glycoprotein, breast cancer resistance p-glycoprotein, and multidrug resistance-associated protein [54]. Moreover, binding of specific plasma proteins to chemotherapeutic drugs has been shown to decrease the concentrations of such drugs and subsequently, delivery of these drugs to the brain [41]. Therefore, poor drug delivery across the BBB is thought to be a major factor underlying the limited efficiency of systemic chemotherapies against BM from breast cancer, particularly monoclonal antibodies like Trastuzumab [48]. Large hydrophilic molecules, such as chemotherapeutic and molecular-targeted drugs, are excluded from the central nervous system unless they can be actively transported by receptor-mediated transcytosis. This highlights the need for new brain-permeable drugs.

In order to metastasize to the brain, breast cancer cells must attach to and invade through the BBB. A widely supported hypothesis is that breast tumour cell adhesion induces retraction of the endothelium, which exposes the vascular basement membrane to the breast cancer cells. There is ample evidence that breast cancer cells recognize and bind to components in the vascular membrane, thereby initiating extravasation and the beginning of colonization at secondary organ sites [43]. The impairment of the BBB was observed recently in breast cancer patients who developed metastasis to the brain. CXCR4/SDF-1*α* has been suggested to play a major role in penetration of breast cancer cells through brain microvascular endothelial cells. In this regard, it has been reported that SDF-1-*α*-mediated blood vessel instability via enhanced vascular permeability facilitates BM from breast cancer in a PI3K/Akt dependent manner [35]. 

The characteristics of the BBB necessitate the use of specialized, biologically relevant (and preferably throughput) preclinical models to test the efficacy of therapeutics against BM. Neural-cancer cell co-cultures can be used to simulate a brain-like microenvironment *in vitro* [55], but these simple models do not test whether compounds could cross the BBB *in vivo*. Bos and colleagues have developed a more biologically relevant *in vitro* transmigration assay, in which cancer cells adhere to and migrate across a human umbilical vein endothelial cell barrier towards human astrocytes segregated in a transwell chamber [34]. The gold standard preclinical model would involve xenograft transplantation of human BM into immunocompromised mice, but development is limited by the availability of fresh human tissue, and to our knowledge, none have been developed using metastatic cells selected in the human brain microenvironment. Alternative models have been generated by *in vivo* selection of neurotropic clones in nude mice, following intracardiac injection of metastatic cells from breast (231-BR, MCF7-HER2-BR, CN34-BM, and the mouse mammary tumour-derived 4T1 syngeneic model) [56, 57].  Table 1 summarizes a series of genes derived from integrated studies that have been implicated in BM development.

## 6. Treatment Strategies against Metastatic Breast Cancers Including Brain Metastases

We are approaching an era of personalized medicine, where therapies will be routinely tailored to individual tumours based on molecular diagnostics. Several targeted therapies for metastatic breast cancer are currently used or in clinical trials (Table 2; examples discussed below).

A recent phase II clinical trial using Lapatinib (dual inhibitor of HER1/2 with good brain penetration) in HER2+ breast cancer patients with BM despite prior radiation and Trastuzumab therapy showed Lapatinib has modest CNS antitumour activity [68]. This was corroborated in a mouse model where Lapatinib inhibited the colonisation of 231BR cells overexpressing EGFR and HER2 [48]. There is also evidence of synergism with Capecitabine, a pyrimidine analogue prodrug approved for metastatic breast cancer management [41, 54]. Sunitinib, a multikinase inhibitor (targets include VEGF-Rs, c-kit and PDGF-Rs), is also under investigation for its effects on BM in metastatic breast and renal cell carcinomas [64, 65, 69].

## 7. Possible “Druggable” Targets for the Future in BM from Breast Cancer

A recent study has demonstrated the role of *STAT3* in BM from breast cancer [38]. Moreover, it has been shown that *STAT3* inhibition by restoration of its inhibitor, suppressor of cytokine signalling (SOCS-1), results in induction of Caveolin-1, a tumour suppressor gene in breast cancer [38]. In this regard, STAT3 might be an attractive therapeutic target in BM from breast cancer. 

Secondly, a growing body of evidence suggests that heparanase, a downstream target of EGFR/HER2, might be involved in BM from breast cancer [53]. EGF induces nucleolar translocation of HPSE and induction of DNA topoisomerase I which is essential for BM from breast cancer and cell proliferation [53]. In this setting, heparanase has been suggested as a potential target that could be exploited therapeutically [53]. 

Palmieri et al. [49] reported that hexokinase 2 (HK2) is significantly increased in brain metastases compared to unmatched primary breast tumors. Hexokinases phosphorylate glucose to produce glucose-6-phosphate in the first step of glucose metabolism. It is thought that HK2 increases glycolysis in tumor cells and may therefore be an attractive therapeutic target [49]. 

We recently identified somatic activating mutations in genes associated with the AKT and MAPK signaling pathways, including *PIK3CA*, *KRAS*, *HRAS*, and *NRAS*, in BM from breast and other cancers [12]. This highlights the possibility of cancer cells resisting targeted treatment to molecules such as HER2 or EGFR by acquiring oncogenic mutations in downstream pathways. Therapeutic modalities targeting these downstream pathways are currently being investigated [59]. In addition, Grigoriadis et al. demonstrated the presence of CT-X antigens in a cohort of BM, including BM from breast cancer. These proteins are predominantly expressed in human germ cells and not somatic tissues, but are frequently activated in cancer. Nearly two-thirds of our BM cohort showed expression of MAGE-A and NY-ESO, two of the CT-X antigens for which inhibitors are currently in clinical trials for lung cancer and melanoma [70]. Targeting these antigens could therefore also be an effective therapeutic strategy for BM.

## 8. Future Directions: Brain Metastases in the Whole Genome Sequencing Era

Direct, high-resolution genomic and transcriptomic analyses of BM may uncover new druggable features that can be targeted to specifically inhibit BM cells. The development and continual refinement of WGS technologies has dramatically increased the resolution with which we can analyse cancer genomes. We can now screen for new mutations, simultaneously and accurately assess their frequencies and correlate this with expression data across the entire genome. Rare mutations, that are present in only a small percentage of cells within the heterogeneous primary tumour mass, may be important in progression and virulence acquisition at a metastatic site. Although there are no large-scale BM genomics studies published to date, Ding and colleagues used WGS to interrogate the genomic profiles of a human BM, its corresponding primary breast tumour, and a mouse xenograft derivative [44]. They showed that; (1) most of the genomic changes present in the primary tumour are propagated during the clinical course of the disease; (2) the frequency of some mutations was increased in the BM, suggesting enrichment with a subpopulation of cells selected from a more heterogeneous primary tumour; (3) two different populations of cells from the primary tumour, with distinct subsets of mutations, were selected during the metastatic process. The third finding is striking and is consistent with other data suggesting that metastases may be established from clusters of cells, rather than one founder cell [71] or may derive from regions of the tumour where there is clonal heterogeneity [16].

## 9. Conclusions

Thus far, we have only fragmented knowledge of the mechanisms underlying breast cancer cell colonisation of the brain microenvironment. Animal models and gene expression profiling have and will continue to provide insight, however it is anticipated that next-generation sequencing technology and integration of this data with expression profiling will enable us to generate a comprehensive map of the brain metastasis genomic landscape. To be able to translate this impending knowledge into clinical outcomes, we must continue to develop and refine high-throughput, biologically relevant preclinical models that accurately mimic the brain microenvironment and the BBB (summarized in Figure 1).

## Figures and Tables

**Table 1:** Genes implicated in the development of brain metastases from breast cancer.

Gene abbrev	Gene name	BM expression status	Gene product functions	Reference
*KCNMA1*	Potassium large conductance calcium-activated channel, subfamily M, alpha member 1	↑	Voltage gated ion channel involved in neuronal excitability	[32]
*MNT*	MAX binding protein	↓	Myc antagonist	[33]
*TERC*	Telomerase RNA component	↑	A template for telomere repeat	[33]
*CTSB*	Cathepsin B	↑	Lysosomal cysteine proteinase	[33]
*PTGS2*	Prostaglandin-endoperoxide synthase 2	↑	Prostaglandin biosynthesis	[34]
*HBEGF*	Heparin-binding EGF-like growth factor	↑	EGFR signalling	[34]
*ST6GALNAC5*	ST6 N-acetylgalactosaminide alpha-2,6-sialyltransferase 5	↑	Sialyltransferase that modifies proteins and ceramides	[34]
*CXCR4*	Chemokine (C-X-C motif) receptor 4	↑	Receptor for stromal cell-derived factor-1	[35]
*MMP2*	Matrix metallopeptidase 2	↑	Degradation of extracellular matrix	[36]
*MMP9*	Matrix metallopeptidase 9	↑	Degradation of extracellular matrix	[36]
*ROBO1*	Roundabout	↑	Axon guidance and neuronal precursor cell migration	[37]
*ERBB3*	Y-erb-b2 erythroblastic leukemia viral oncogene homolog 3	↑	Cell proliferation and differentiation	[12]
CAV1	Caveolin 1, caveolae protein	↓	Structural component of the caveolae plasma membranes	[38]
*ERBB2*	v-erb b2 erythroblastic leukemia viral oncogene homolog 2	↑	Cell proliferation	[39]
HK2	Hexokinase 2	↑	Glycolysis	[34]
HPSE	Heparanase	↑	Remodeling of the extracellular matrix	[40]
STAT3	Signal transducer and activator of transcription 3	↑	Cell growth and apoptosis, inflammation, invasion, and metastasis	[39]
VEGF	Vascular endothelial growth factor	↑	Stimulates angiogenesis and vasculogenesis	[41]
IL-8	Interleukin 8	↑	CXC chemokine involved in neutrophil recruitment	[41]

↑  overexpressed*; *↓  downregulated.

**Table 2:** Current targeted therapies and their use in metastatic breast cancer (MBC).

Drug	Class	Targets	Clinical indications	Reference
Trastuzumab	mAb	HER2	Single agent for HER2+ MBC; used in combination with paclitaxel as first-line therapy for HER2+ MBC	[58]

Lapatinib	TKI	HER1/2	Active in Trastuzumab-resistant, HER2+ breast cancer; crosses the BBB and suppresses CNS metastasis used in combination with Capecitabine for HER2+ MBC	[41]

Pertuzumab	mAb	HER2	Impairs HER2 homo-/hetero-dimerisation; active in Trastuzumab-resistant HER2+ breast cancers; not currently approved for MBC	[59]

Neratinib	TKI	HER1/2	Inhibits HER2 autophosphorylation and suppresses downstream signalling; active in HER2+ patients with and without Trastuzumab pretreatment; not currently approved for MBC	[60]

Bevacizumab	mAb	VEGF	Antiangiogenic therapy for MBC in combination with Docetaxel or Paclitaxel for first-line treatment	[61]

Sorafenib	TKI	VEGFR PDGFR Raf	Multitarget receptor tyrosine kinase inhibitor; FDA-approved for advanced renal cancer and hepatocellular carcinoma	[62]

Axitinib	TKI	VEGFR PDGFR	Currently in phase III clinical trial for metastatic renal cell carcinoma	[63]

Sunitinib	TKI	VEGFR PDGFR KIT	Antiangiogenic therapy; FDA-approved for renal cell carcinoma and Gleevec-resistant gastrointestinal stromal tumours; effective as a single agent for metastatic breast cancer (phase II study)	[64–66]

Pazopanib	TKI	VEGFR PDGFR KIT	Anti-angiogenic therapy; FDA-approved for renal cell carcinoma; combination with Lapatinib has superior activity as the first-line treatment for MBC (phase II study)	[67]

Abbreviations: BBB: blood-brain barrier; CNS: central nervous system; HER1: Epidermal Growth Factor Receptor (EGFR); mAb: monoclonal antibody; KIT: Mast/stem cell growth factor receptor gene; PDGFR: platelet-derived growth factor receptor; TKI: tyrosine kinase inhibitor; VEGFR: vascular endothelial growth factor receptor.
